# The Evolution and Origin of Animal Toll-Like Receptor Signaling Pathway Revealed by Network-Level Molecular Evolutionary Analyses

**DOI:** 10.1371/journal.pone.0051657

**Published:** 2012-12-07

**Authors:** Xiaojun Song, Ping Jin, Sheng Qin, Liming Chen, Fei Ma

**Affiliations:** 1 Laboratory for Comparative Genomics and Bioinformatics & Jiangsu Key Laboratory for Biodiversity and Biotechnology, College of Life Science, Nanjing Normal University, Nanjing, People’s Republic of China; 2 The Key Laboratory of Developmental Genes and Human Disease, Ministry of Education, Institute of Life Science, Southeast University, Nanjing, Republic of China; CNRS, France

## Abstract

Genes carry out their biological functions through pathways in complex networks consisting of many interacting molecules. Studies on the effect of network architecture on the evolution of individual proteins will provide valuable information for understanding the origin and evolution as well as functional conservation of signaling pathways. However, the relationship between the network architecture and the individual protein sequence evolution is yet little known. In current study, we carried out network-level molecular evolution analysis on TLR (Toll-like receptor ) signaling pathway, which plays an important role in innate immunity in insects and mammals, and we found that: 1) The selection constraint of genes was negatively correlated with its position along TLR signaling pathway; 2) all genes in TLR signaling pathway were highly conserved and underwent strong purifying selection; 3) the distribution of selective pressure along the pathway was driven by differential nonsynonymous substitution levels; 4) The TLR signaling pathway might present in a common ancestor of sponges and eumetazoa, and evolve via the *TLR*, *IKK*, *IκB* and *NF-*κ*B* genes underwent duplication events as well as adaptor molecular enlargement, and gene structure and conservation motif of *NF-κB* genes shifted in their evolutionary history. Our results will improve our understanding on the evolutionary history of animal TLR signaling pathway as well as the relationship between the network architecture and the sequences evolution of individual protein.

## Introduction

Genes perform their biological functions within genetic pathways via interacting with other molecules in networks [1], [2]. It may provide important insights into the evolutionary constraints of genes in molecular networks via establishing the genetic variation pattern of genes across networks and revealing the impact of natural selection on such variability. Many studies have shown that proteins in the center of networks experienced strong evolutionary constraints, while proteins in the periphery of networks seemed to undergo positive selection, and physically interacting proteins in networks showed similar evolutionary rates [3], [4], [5], [6]. The distribution of gene selective pressure within networks might depend on network function [7]. However, it’s still unclear how the network architecture impacts on gene evolution in networks. Comparison of the nucleotide substitution patterns in multiple networks would possibly discover general rules for gene evolution within genetic networks.

Downstream genes experienced relaxed selection pressure and evolved faster than upstream ones do in several pathways, such as the anthocyanin pathway [8], [9], [10] and the carotenoid & terpenoid biosynthetic pathway in plants [11], [12], and the melanin synthesis pathway in silkworms [13]. In these pathways, a gene nucleotide substitution rate always positively correlates to the gene position in network. In contrast, downstream genes tend to evolve more slowly than upstream ones do in the insulin/Tor signaling pathway in Drosophila and vertebrates [2], [14]. In the insulin/Tor signaling pathway in *Caenorhabditis*, there is no relationship between the rate of nonsynonymous/synonymous substitution and the position of a gene in network [1], while in the HOG-signaling pathway in yeast [15] and the N-Glycosylation metabolic pathway across primates [16] there is a negative correlation between the nucleotide substitution rate and position of a gene in network.

The TLR signaling pathway, which plays a central role in innate immunity from Drosophila to mammal [17], is highly conserved in structure and function from insects to vertebrates [18]. Many immunomodulatory properties of the TLR signaling pathway have been found in some early metazoan genomes such as *Nematostella vectensis* [19], *Trichoplax* [20] and *Amphimedon queenslandica* [21]. Surveying early-branching metazoans (*Nematostella vectensis*), and the recent discovery of unusual *TLR*-like genes in various multicellular animals (*Amphimedon queenslandica* and *Hydra magnipapillata*) [22], [23], [24] would help us to decipher the origin of this toll-like receptor superfamily. Although the *NF-κB* gene was detected in single-celled eukaryote *Capsaspora owczarzaki*, the toll-like receptor was not found in single-celled eukaryotes [25], and the *NF-κB* gene is lost in *Caenorhabditis elegans* [26]. The evolution history of the animal TLR signaling pathway is still unknown.

**Table 1:** The genes of the TLR signaling pathway.

gene	position	protein length	connectivity	ENC	% used coden	*dn*	*ds*	*ω*
*TLR1*	1	786	9	53.65	80.14	1.45	6.76	0.22
*TLR2*	1	784	18	53.95	94.69	1.42	5.99	0.24
*TLR3*	1	904	6	54.08	95.18	1.04	6.42	0.16
*TLR4*	1	839	22	53.78	81.23	2.08	7.29	0.29
*TLR5*	1	858	5	54.41	86.89	1.47	7.22	0.20
*TLR6*	1	796	3	52.10	95.24	0.78	3.18	0.24
*TLR7*	1	1049	1	53.27	98.21	0.60	3.75	0.16
*TLR8*	1	1041	2	56.75	93.64	1.12	4.83	0.23
*TLR9*	1	1032	0	42.94	90.91	1.32	9.29	0.14
*MyD88*	2	317	24	47.24	70.20	0.74	7.48	0.10
*TIRAP*	2	221	14	45.99	44.77	1.49	9.03	0.17
*TRAM*	2	235	0	54.23	97.45	0.24	1.03	0.23
*TRIF*	2	712	20	47.77	63.45	1.70	10.03	0.17
*TOLLIP*	2	274	18	43.77	59.17	0.34	6.41	0.05
*IRAK1*	3	682	35	45.46	17.68	1.98	8.55	0.23
*IRAK4*	3	460	7	49.63	59.62	1.04	8.46	0.12
*TRAF6*	4	522	303	55.21	91.28	0.63	8.45	0.07
*TRAF3*	4	568	41	50.62	89.55	0.50	14.01	0.04
*TAB1*	5	504	13	47.06	52.35	0.40	8.76	0.05
*TAB2*	5	693	13	52.61	93.13	0.18	2.14	0.09
*TAK1*	5	579	0	55.87	30.50	0.04	4.91	0.01
*RIPK1*	4	671	0	52.79	62.46	1.36	6.87	0.20
*IKKα*	6	745	7	52.84	90.62	0.42	7.30	0.06
*IKKβ*	6	754	52	48.56	49.07	0.70	7.80	0.09
*IKKγ*	6	487	46	46.90	52.58	0.78	13.40	0.06
*IKKζ*	6	631	271	44.71	37.77	0.58	7.87	0.07
*TBK1*	6	729	17	50.34	99.18	0.40	8.95	0.04
*MEK1*	6	393	33	51.21	98.99	0.12	7.85	0.02
*MEK2*	6	400	17	43.04	71.32	0.13	4.04	0.03
*MKK3*	6	318	12	41.53	74.35	0.05	4.89	0.01
*MKK6*	6	334	15	52.56	91.76	0.13	5.37	0.02
*MKK4*	6	399	24	53.89	80.36	0.11	3.06	0.03
*MKK7*	6	419	17	45.58	67.25	0.24	6.11	0.04
*IKBα*	7	317	62	49.01	92.64	0.77	12.87	0.06
*MAPK1*	7	360	176	54.63	93.77	0.03	10.73	0.00
*MAPK3*	7	357	113	42.97	77.21	0.17	2.69	0.06
*MAPK11*	7	364	15	46.54	95.60	0.25	10.57	0.02
*MAPK12*	7	367	18	44.31	64.72	0.43	8.22	0.05
*MAPK13*	7	365	9	44.18	88.30	0.50	7.92	0.06
*MAPK14*	7	360	81	52.03	99.72	0.17	6.29	0.03
*MAPK8*	7	384	68	54.79	89.23	0.14	5.10	0.03
*MAPK9*	7	424	38	54.63	69.18	0.10	13.22	0.01
*MAPK10*	7	422	15	53.75	79.50	0.04	4.09	0.01
*p105*	8	968	72	54.17	78.79	0.37	3.73	0.10
*p65*	8	548	109	45.88	69.69	0.80	15.55	0.05
*IRF5*	8	498	6	43.72	69.37	0.48	9.77	0.05
*IRF7*	8	503	12	43.59	55.52	1.70	12.26	0.14
*IRF3*	8	452	16	48.15	43.31	1.43	6.04	0.24
*FOS*	8	380	52	44.18	83.29	0.57	11.50	0.05
*JUN*	8	331	111	42.17	82.39	0.21	7.80	0.03

Here, we study the evolution and origin of TLR signaling pathway in animals at network-level. Our results showed that the nucleotide substitution rate was negatively correlated with gene position along TLR signaling pathway from receptors to transcription factors, and all genes underwent relatively strong purifying selection. We also found that selective pressures on genes along the pathway were driven by nonsynonymous substitutions. More importantly, we provided evidences to support that the TLR signaling pathway was presented in Porifera, but not in choanoflagellate *Monosiga brevicollis*, *Saccharomyces cerevisiae* and *Caenorhabditis elegans*. In addition, *NF-κB* genes underwent strong selection pressure in the evolution and showed positive selection in some branches of the evolutionary tree, which might be connected with the gene duplication, gene structure shift and domain lost. Our findings suggested that the TLR signaling pathway might present in a common ancestor of eumetazoa, such as sponge and placozoans.

**Figure 1:**
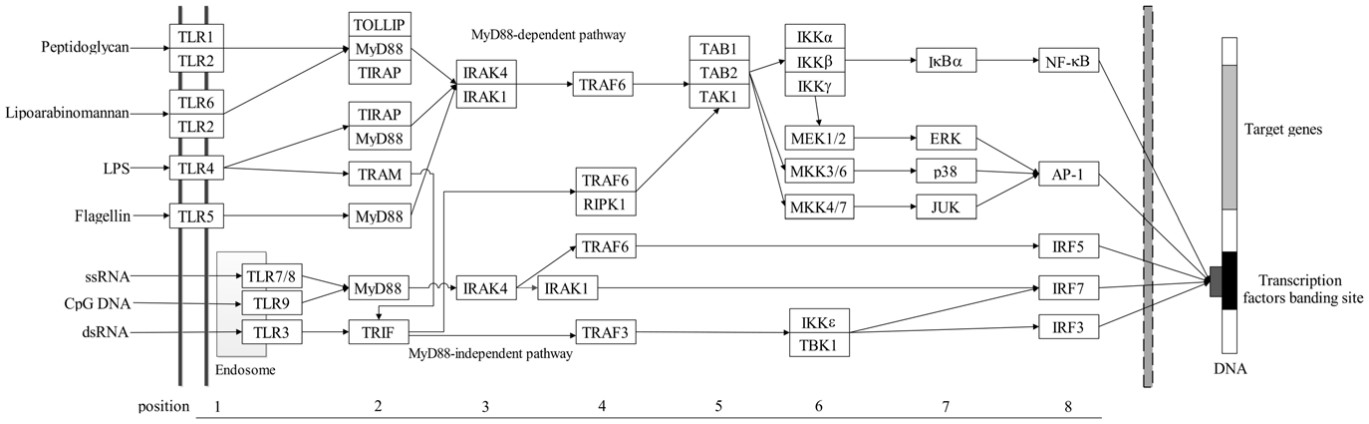
The TLR signaling pathway genes in vertebrate. Arrows indicate the direction of signal transduction. The numbers on the below side represent the position of the TLR signaling pathway genes. This picture is adapted from KEGG database.

**Figure 2:**
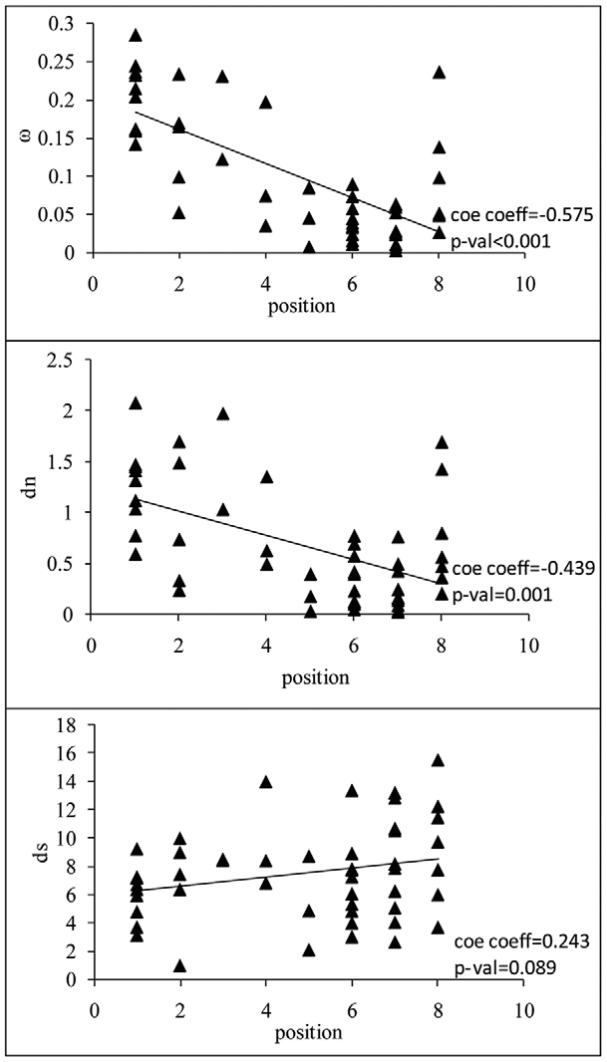
The nonsynonymous/synonymous substitution rate ratio (*ω* = *dn/ds*), nonsynonymous substitution rate (*dn*), and synonymous substitution rate (*ds*) versus pathway position. The correlations are significant for *ω* and *dn*. This shows that the strength of the purifying selection increases from upstream to downstream.

**Table 2:** Bivariate Correlations.

		position	*ω*	*dn*	*ds*	ENC	% used coden	connectivity	protein length
position	ρ		−0.575^**^	−0.439^**^	0.243	−0.321^**^	−0.15	0.479^**^	−0.447^**^
	*P*		<0.001	<0.001	0.089	0.02	0.30	<0.001	<0.001
*ω*	ρ	−0.575^**^		0.849^**^	−0.16	0.10	−0.07	−0.331^*^	0.493^**^
	*P*	<0.001		<0.001	0.28	0.47	0.65	0.019	<0.001
*dn*	ρ	−0.439^**^	0.849^**^		0.305^*^	−0.08	−0.21	−0.20	0.480^**^
	*P*	<0.001	<0.001		0.03	0.59	0.13	0.16	<0.001
*ds*	ρ	0.24	−0.16	0.305^*^		−0.306^*^	−0.21	0.285^*^	−0.08
	*P*	0.089	0.28	0.03		0.03	0.14	0.045	0.59
ENC	ρ	−0.321^*^	0.10	−0.08	−0.306^**^		0.372^**^	−0.11	0.358^*^
	*P*	0.02	0.47	0.59	0.03		0.01	0.45	0.01
% used coden	ρ	−0.15	−0.07	−0.21	−0.21	0.372^**^		−0.08	0.07
	*P*	0.30	0.65	0.13	0.14	0.01		0.60	0.61
connectivity	ρ	0.479^**^	−0.331^*^	−0.20	0.285^*^	−0.11	−0.08		−0.308^*^
	*P*	<0.001	0.019	0.16	0.045	0.45	0.60		0.03
protein length	ρ	−0.447^**^	0.493^**^	0.480^**^	−0.08	0.358^*^	0.07	−0.308^*^	
	*P*	<0.001	<0.001	<0.001	0.59	0.01	0.61	0.03	

## Materials and Methods

### Data collection

The data set contained the TLR signaling pathway-related genes from eight vertebrates. These genes and their interaction networks were downloaded from the KEGG database (PATHWAY: map04620). Here, we mainly focused on these genes involved in TLR signaling pathway. We downloaded these protein coding sequences (CDS) and protein sequences of *Homo sapiens* (human), *Pan troglodytes* (chimpanzee), *Macaca mulatta* (macaque), *Mus musculus* (mouse), *Bos taurus* (cow), *Gallus gallus* (chicken), *Xenopus tropicalis* (western clawed frog), *Danio rerio* (zebrafish) from KEGG database. Finally, the dataset was composed of 50 genes listed in Table 1 and Table S1.

**Figure 3:**
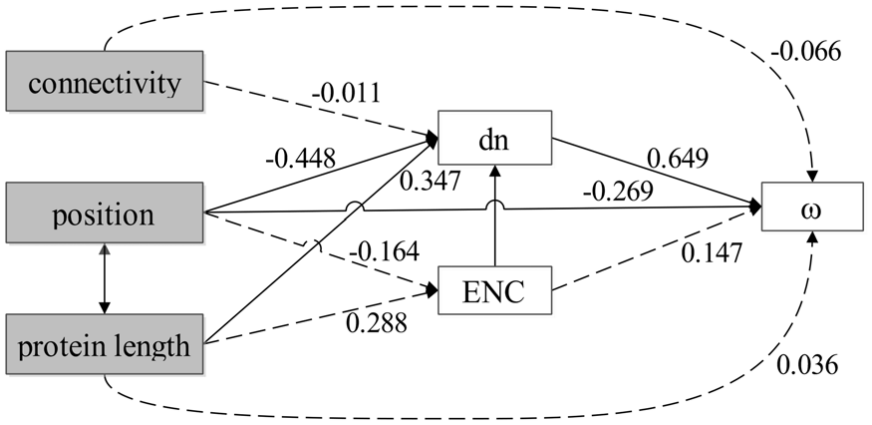
The relationships among element positions in the TLR signaling pathway, protein length, connectivity, nonsynonymous divergence (***dn***)**,** ***dn/ds*** **ratio** (***ω***)**, and codon bias** (**measured by the ENC**)**.** Pathway position, protein length, and connectivity were treated as exogenous variables, while the rest were treated as endogenous variables. The causal dependencies between variables assumed in the model are represented by single-headed arrows. Correlations between exogenous variables are represented by double-headed arrows. The numbers on the arrows represent the standardized path coefficients (β). Solid and broken lines represent significant and nonsignificant relationships, respectively.

However, some sequences from non-human vertebrates showed long deletions in the middle of the genes when aligned to their orthologous group, were likely to be artifacts due to a low quality of the sequence or of the annotation. The putative missing parts of incomplete CDS were recovered through a similarity-search-based procedure as follows: first, a BLAT search against the whole genome of interest on the UCSC genome browser (http://genome.ucsc.edu/cgi-bin/hgBlat) was performed; if not successful, a BLAST search against NCBI Traces (http://blast.ncbi.nlm.nih.gov/Blast.cgi) or Ensemble genome browser (http://www.ensembl.org/info/about/species.html) of whole genomes was performed. When a homologous genomic region could be retrieved, the structure of the gene was predicted with the Wise2 program of the GeneWise tool [27] applied with default options. This program was used to predict the structure of a gene (introns-exons) given a genomic DNA region and a protein sequence of high homology to the putative one. The genomic DNA identified through the BLAT search and the protein sequences of the human reference genes were given as input to the Wise2 which predicted the gene structure. Only good predictions in which there were no internal stop codons or frame shifts were accepted. The longest transcript of a gene with more than one transcription was chosen for analysis (detailed information Table S1).

**Figure 4:**
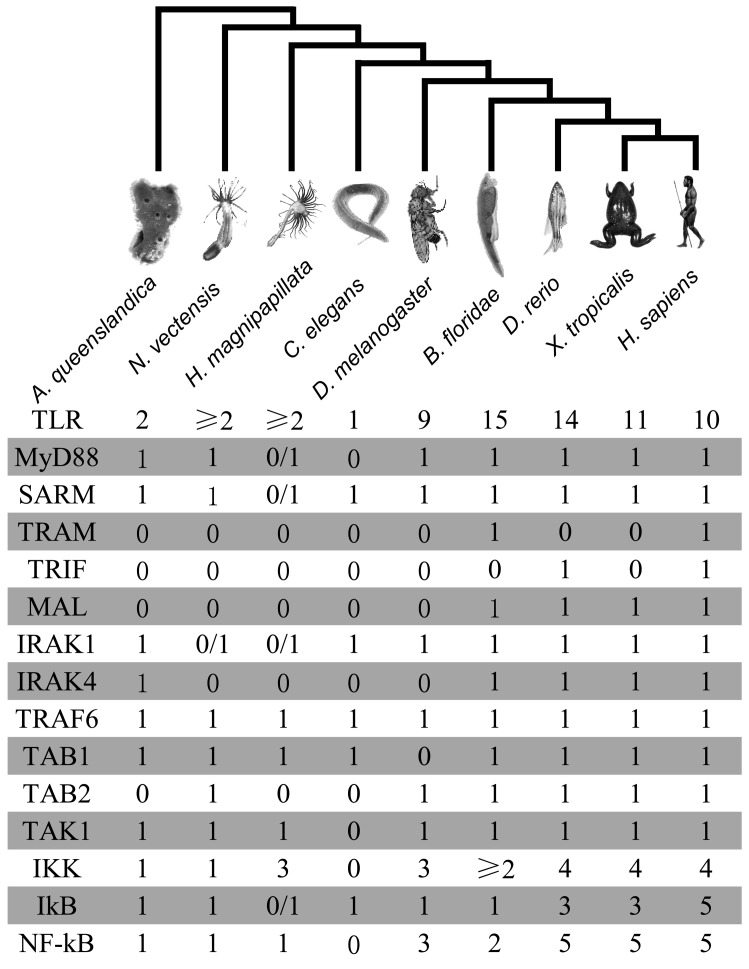
The number of the core components of the TLR signaling pathway in representative metazoans. The numbers represent the copy number of the TLR signaling pathway genes in all studied species. “0/1” means not found using blast research, but it present in lower animals.

**Figure 5:**
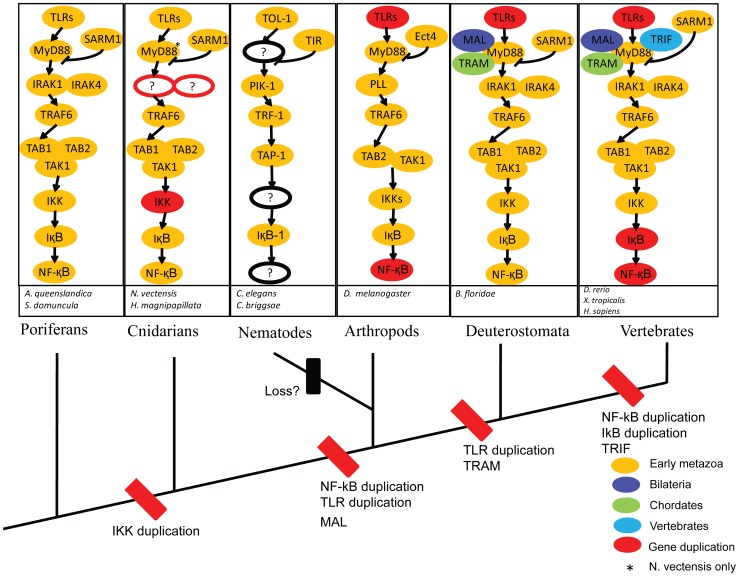
Summary of the TLR signaling pathway evolution. Orthologs between different organisms are placed in the same relative position. If orthologs were not found, the ortholog place marked a question mark (?) with a circle. Divergence times of major metazoa groups are presented next to the relevant branches. Our result shows that the complete core pathway known from bilaterians is represented in the sponge *Amphimedon queenslandica*. The pathway is mostly absent in non-metazoans, *Saccharomyces cerevisiae* and *Monosiga brevicollis*. The nematode *Caenorhabditis elegans* and its relatives *Caenorhabditis Brigssae* are deficient in several of the pathway components. This may indicate of a functional loss of major components in nematodes.

Moreover, to explore the evolutionary origin of the TLR signaling pathway, tblastn and blastp were employed to search the orthologs genes of TLR signaling pathway in genomes of single-celled eukaryotes (*Saccharomyces cerevisiae*, *Monosiga brevicollis*), sponges (*Amphimedon queenslandica*), sea anemone (*Nematostella vectensis*), hydra (*Hydra magnipapillata*), nematode (*Caenorhabditis elegans*), fruit fly (*Drosophila melanogaster*), amphioxus (*Branchiostoma floridae*), zebrafish (*Danio rerio*), frog (*Xenopus tropicalis*), and human (*Homo sapiens*) and so on. All human sequences of TLR signaling pathway-related genes were searched against the Refseq protein data from different organisms. All blast hits were filtered, and only sequences with blast score >150 and length >50 were examined. After that, sequences were tested using reciprocal blast search. A gene was assigned as a homologous gene if the best hit of one blast search matched the best hit of the other. If the sequences were not found reciprocally in two genomes, a gene was assigned as a homologous gene if it is with the best coverage, with score >150, relative identity >30% and relative similarity >40%. If the parameters of a protein were lower or domains of a protein were not similar to its Refseq protein, we would assign it as a “non homologous gene”. When we did not find an ortholog, we verified the lack of orthologous sequences with tblastn against the genome sequence and EST libraries of the relevant organism [28], [29]. These above dates have been shown in Table S3.

**Figure 6:**
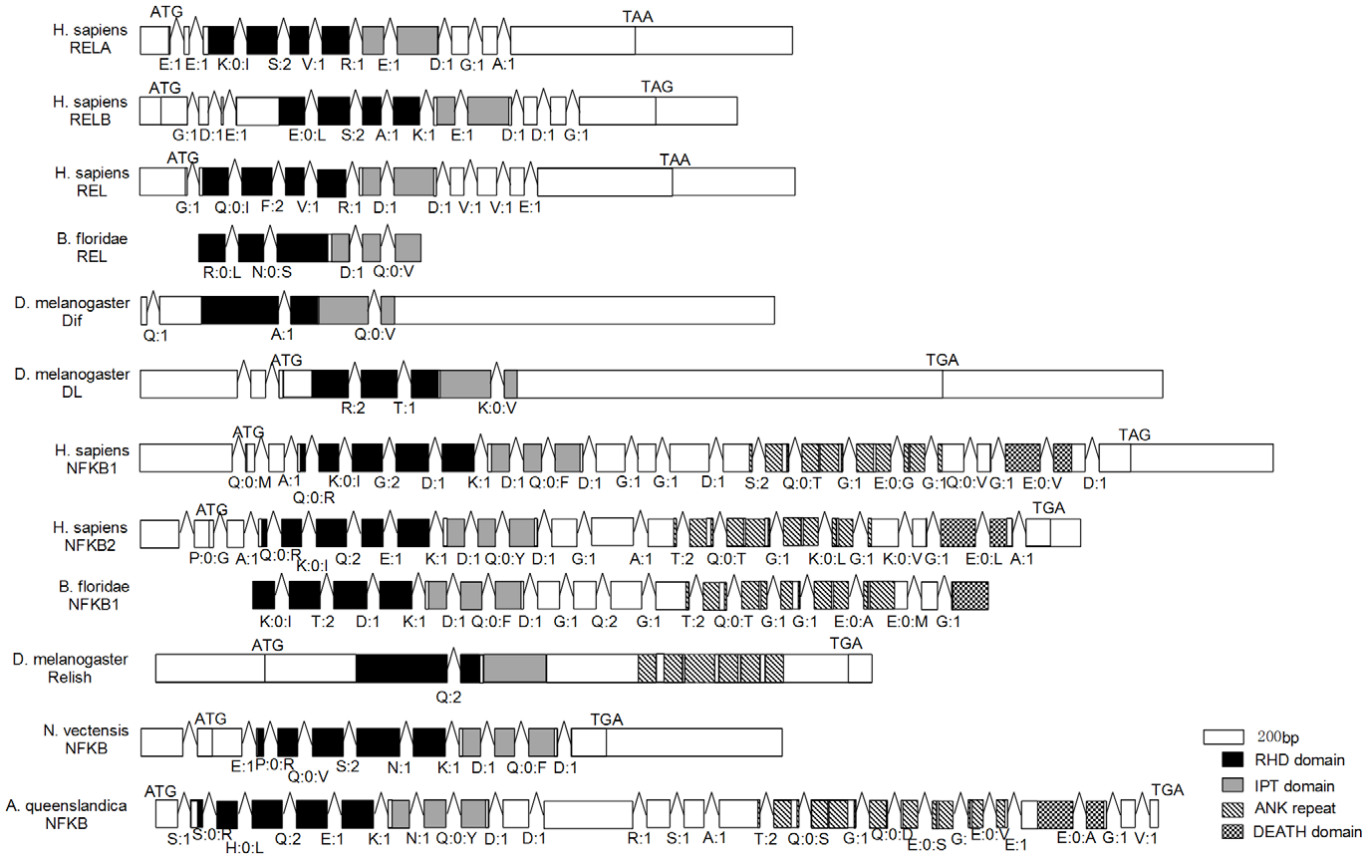
Gene structure and conservation motifs of NF-κB family genes. Major domains are marked with different colors and shapes. The transcript coding region is drawn to scale and introns positions are illustrated but their size is not drawn to scale. The letters and number under the introns represents introns phase.

### Multiple Sequence Alignment and phylogenetic analysis

Multiple sequence alignment was performed for orthologs sequences of TLR signaling pathway using MUSCLE [30] with default parameters, which were manually curated if necessary. Gene structure and position of motifs were checked by hand using data from Entrez gene, and conservation of motifs was predicted by SMART software (http://smart.embl-heidelberg.de/). Phylogenetic analysis was conducted using MrBayes [31] for bayes analysis with mixed amino acid substitution model, and using PhyML [32] for ML analysis with default parameters. Phylogeny support was verified with the bootstrap consensus tree inferred from 1000 replicates.

### Codons-Based sequence Analysis

Nonsynonymous/synonymous substitution rate ratio (*ω* =  *dn*/*ds*) can provide a measurement for the change of selective pressures. Respectively, *ω* = 1, <1 and >1 will indicate neutral evolution, purifying selection, and positive selection on the target gene. Here, we used a codon-substitution model implemented in the CODEML program in the PAML4.4 software package [33] to analyze changes of selective pressure, which allow for variable selection patterns among amino acid sites, M0 (one ratio), M1a (nearly neutral), M2a (positive selection), M7 (beta), M8 (beta & ω), M8a to test for the presence of sites under positive selection. We further tested for the presence of codons evolving under positive selection by contrasting the M1a and M2a models, and the M7 and M8, M8 and M8a models by the likelihood ratio tests (LRTs). Otherwise, to explore the divergence of different branch of NF-κB in the evolution history, we also used branch model of codeml soft to compute the nonsynonymous/synonymous substitution rate ratio of different branches.

### Multivariate analysis

To analyze the evolution of each gene within the context of the structure in the network, we computed differently topological parameters and explored their correlation with the evolutionary rates of genes given by model M0. Some studies indicated that selective constraint levels of the different position of the biology networks might be affected by different factors, including protein length [34], connectivity [4], codon bias [35], and so on. Thus, we performed a multivariate analysis considering *dn*, *ω*, the pathway position, and some other parameters influencing selection levels (codon bias, protein length and connectivity). First, we evaluated whether there is significant correlation among these parameters using Spearman’s rank correlation coefficient (ρ). Then, we analyzed the data using partial correlations and path analysis. Path analysis is an extension of multiple regression analysis that allows decomposing the regression coefficients into their direct and indirect components by considering an underlying user-defined causal model, and to assess the statistical significance of the relevantly direct components. We therefore performed path analysis to find which is the main factor influencing the trends of the *ω* values. Connectivity, pathway position, protein length were considered as exogenous variables, whilst the ENC (effective number of codons ), *dn* and *ω* were considered as endogenous variable. All these analyses were conducted using the PASW statistical software. Connectivity was estimated by the number of PPIs which data from the human interaction network of Bossi and Lehner [36]. The codon usage bias of each orthologous group gene was measured by the median of the ENC across all studied species. ENC values were computed using the DnaSP 5.10.01 software [37].

## Results and Discussion

### Analysis of the evolutionary rates

The selection pressures acting on genes can be inferred by nonsynonymous/synonymous substitution rates. For each gene in the TLR signaling pathway, the M0 model estimated a single nonsynonymous/synonymous substitution rate (*ω* = *dn/ds*) for all lineages. The *ω* values of genes in the TLR signaling pathway ranged from 0.00721 (*MAPK9*) to 0.25817 (*TLR4*) using the M0 model (Table 1). These results indicated that TLR signaling pathway-related genes have undergone strong purifying selection with strong functional constrains. In addition, we found the variation pattern along the TLR signaling pathway was unlikely to predominantly result from positive selection acting on the upstream genes (Table S2), but may be caused by increasing levels of purifying selection [1].

### The strength of the selective constraints and pathway structure

Since genes in the TLR signaling pathway had a clear sequentiality (Fig.1), the correlation between the evolutionary rate of a gene and its position in the pathway was tested. The results demonstrated that the *ω* value of a gene was strongly negatively correlated with its position in the pathway (Spearman’s rank correlation coefficient: ρ = −0.575, *P*<0.001; Table 2 and Fig. 2), and indicated that the downstream genes evolved more slowly than the upstream ones did. Similar results were also observed along the insulin/Tor pathway in Drosophila [2], and in vertebrates [14], as well as along the N-Glycosylation metabolic pathway in primates [16]. Our findings suggested that the topological structure of the TLR signaling pathway influenced the distributions of selective constraints of genes. One possible explanation was that in signal transduction pathway, evolutionary changes might preferentially localize to the receptor which interacted with the external environment of cells, while downstream elements which were located within a more stable cytoplasmic milieu might be expected to be under stronger purifying selection. Consistently, a global analysis of human-signaling pathways showed that purifying selection increased from the extracellular space to the nucleus [38].

Analyses were further performed to determine the relationship between the nonsynonymous substitution rate (*dn*) and synonymous substitution rate (*ds*) of genes and their position in the pathway. Spearman rank’s correlation analysis showed that the gene position was significantly correlated with the *dn* (*r* = 0.439, *P*<0.001), but non-significantly correlated with the *ds* (*r* = 0.243, *P* = 0.089). The results implied that nonsynonynous substitution rate changes might be the main contributor to the above negative tendency.

The selective constraints on the pathway genes could be affected by other factors. The association between evolution rate of gene in the TLR signaling pathway with its codon usage, connectivity and protein length were explored (Table 2). A simple Spearman’s rank correlation analysis showed that the *ω* value was, respectively, significantly negatively correlated with connectivity (ρ = −0.331, *P* = 0.019), and significantly positively correlated with protein length (ρ = 0.493, *P*<0.001), whereas non-significantly correlated with codon usage (ρ = −0.07, *P* = 0.65).

In addition, as shown in Table 2, the position of gene in the TLR signaling pathway was significantly correlated with the *ω* value, *dn* (ρ = −0.439, *P*<0.001), codon bias (ρ = −0.321, *P* = 0.02), protein length (ρ = −0.447, *P*<0.001) and connectivity (ρ = 0.479, *P*<0.001). And the *dn* vaule was significantly correlated with protein length (ρ = 0.48, *P*<0.001). The *ds* vaule was also significantly negatively correlated with codon bias (ρ = −0.306, *P* = 0.03), and significantly positively correlated with connectivity (ρ = 0.285, *P* = 0.05). The protein length was significantly positively correlated with codon bias (ρ = 0.358, *P* = 0.01), and significantly negatively correlated with connectivity (ρ = −0.308, *P* = 0.03).

### Multivariate analysis

To clarify whether the observed correlations resulted from indirect or direct effects, two multivariate analysis techniques were applied (partial correlation analysis and path analysis) to evaluate the association between the pathway position and *ω*, *dn*, and *ds* values controlling for the factors discussed above. Partial correlation revealed that when controlling for gene position, the correlation between connectivity and *ω* vaule was non-significant (*r* = −0.103, *P* = 0.48), but the correlation between position and *ω* was still significant when controlling for connectivity (*r* = −0.676, *P*<0.001). Similarly, when controlling for ω, the correlation between position and connectivity was non-significant (*r* = 0.136, *P* = 0.353), the correlation between *dn* and position was also non-significant (*r* = 0.095, *P* = 0.516). Instead, the correlation between *dn* and *ω* holded significant when controlling for the position and connectivity (*r* = 0.738, *P*<0.001). This result supported that the main observed effect was the correlation between *dn* and *ω*. The *dn* and position of genes in TLR signaling pathway were very important factors affecting selection pressure of TLR signaling pathway-related genes.

Recent studies showed that protein length was not the main factor to affect the pathway evolution process [2], [14]. However, as shown in table 2, protein length was significantly correlated with all other factors except *ds*. To address this issue, partial correlation analysis was applied to evaluate the association between protein length and other factors. When controlling for *ω*, the protein length was not correlation with position (*r* = −0.215, *P* = 0.138), *dn* (*r* = 0.05, *P* = 0.735), and connectivity (*r* = 0.001, *P* = 0.995). Similarly, when controlling for connectivity, codon bias, position, respectively, the correlation between *ω* and protein length consistently showed significant. Therefore, the *ω* was a main factor in our study, and the protein length could correlate with other factors indirectly. Recently, many studies showed that protein length appeared to be an important factor virtually influencing all aspects of molecular evolution [34], [39], [40]. Thus, protein length may influence the evolution of the TLR signaling pathway of vertebrate.

To better characterize the relationships within these variables, a path analysis under the model was performed and the result was presented in Figure 3. The path analysis revealed that the *ω* value was affected by the *dn* and position of the gene in the pathway (standardized path coefficient, *dn*: β = 0.649, *P*<0.001; position: β = −0.269, *P* = 0.006), even after removing the effects of putatively relevant factors (codon bias and protein length). The *dn* value was positive associated with protein length (β = 0.347, *P* = 0.016) and negative associated with position (β = −0.448, *P* = 0.002) and codon bias (β = −0.290, *P* = 0.026). And the protein length could affect the *ω* value through affecting the *dn* value. Among the features that described the network, the *dn* was the main factor shaping the rate of the natural selection. Nevertheless, these factors affecting protein evolution in TLR signaling pathway were complex, with interplay of many factors.

### The evolutionary origin of the NF-κB-mediated TLR signaling pathway

TLR signaling pathway played an important role not only in innate immunity in vertebrate and insect, but also in development of embryos dorso-ventral pattern at Drosophila [18], [41]. However, the evolutionary origin of the TLR signaling pathway is still unknown. To explore the evolutionary origin of the TLR signaling pathway, blastp and tblastn were employed to search the orthologs genes of TLR signaling pathway in genomes of single-celled eukaryotes (*Saccharomyces cerevisiae*, *Monosiga brevicollis*), sponges (*Amphimedon queenslandica*), sea anemone (*Nematostella vectensis*), hydra (*Hydra magnipapillata*), nematode (*Caenorhabditis elegans*), fruit fly (*Drosophila melanogaster*), amphioxus (*Branchiostoma floridae*), zebrafish (*Danio rerio*), frog (*Xenopus tropicalis*), and human (*Homo sapiens*). Compared to *NF-κB* which was not been found in prokaryote [25], the MAPK signaling pathway was involved in invasive growth and detected in prokaryote [42]. Thus, MAPK signaling pathway was not taken into account.

Figure 4 and Figure 5 depicted the composition of evolution history of the TLR signaling pathway from sponges to vertebrates. In *Saccharomyces cerevisiae* and *Monosiga brevicollis* genomes, homologous of the core pathway were not found, such as toll-like receptor genes and *NF-κB* genes (data not shown). Therefore, convincing evidence was lacking for the TLR signalling pathway in single-celled eukaryotes. Recently studies showed that A20/AN1 zinc-finger domain-containing family proteins were well characterized and known to play a central role in regulating the immune response in animals and responding to different types of stresses including cold, desiccation, salt, submergence, heavy metals, wounding, and the stress hormone in plants [43], [44], 45. Interestingly, three *A20/AN1* genes were found in *Monosiga brevicollis* [46], suggesting *Monosiga brevicollis* could be resistant to pathogen infections. Meanwhile, a single *C1qDC* gene, which was regarded as a major connecting link between innate and acquired immunity, and Tyrosine kinases (TyrK) proteins, which was essential for cell–cell communication in animals, mediating hormone, growth factor, immune, and adhesion-based signaling, had been detected in the choanoflagellate *Monosiga brevicollis* genome [47], [48].

In the metazoan clade, a relatively complete TLR signaling pathway was identified in *Amphimedon queenslandica* genome, including some core genes of pathway, such as 2 *TLR*s, *MyD88*, *SARM1*, *TRAF*, *IKK*, *IκB* and *NF-κB*. This finding suggested that the TLR signaling pathway might origin from early metazoan. Interestingly, the domain analysis showed that TLRs in sponge contained a TIR domain, a transmembrane anchor and two Ig domains. Unlike vertebrates in which TIR and LRR domains were found in a single protein sequence of TLRs, *Amphimedon queenslandica* genome codes the TIR and LRR domains were not detected in a single protein sequence [23]. However, *SDTLR*, a *TLR*-like gene in the sponge *Suberites domuncula* [49], only had a TIR domain and a transmembrane anchor, and contained no Ig and LRR domains. Therefore, those *TLR*-like genes might be the ancestor gene of *TLR*s in the evolution history. Otherwise, the gene structure and motifs analysis of Rel/NF-κB family showed that NF-κB protein had 7 ANK repeats in the *Amphimedon*, 6 ANK repeats in vertebrates and none ANK repeats in sea anemone (Fig 6).

Similar TLR signaling pathway topology was found between *Nematostella vectensis* and *Hydra magnipapillata*. The cnidarians *Nematostella vectensis* is considered to be the closest outgroup to the bilaterians among the known extant taxa [50], [51]. At least two *TLR*-like genes were detected in *Nematostella vectensis* and *Hydra magnipapillata*. Like the *TLR*-like genes in *Amphimedon queenslandica*, the *TLR*-like genes in *Nematostella vectensis* and *Hydra magnipapillata* had a TIR domain and a transmembrane anchor without any LRR domains. Unexpectedly, *IRAK* orthologous was not detected in two animals: *Nematostella vectensis* and *Hydra magnipapillata*. This phenomenon might be due to either the too low identity to be detected between the *IRAK*-like gene in these two animals and human *IRAK* genes using reciprocal blast, or incompleteness of gene ontology of these two animals’ genomes or incompleteness of the genomes of these two animals. The *IKK* (I kappa B kinase) gene had gene duplication in *Hydra magnipapillata* (Fig. 4 and Fig. 5). Interestingly, although *NF-κB* contained no ANK repeats and Death domain in *Hydra magnipapillata* and *Nematostella vectensis*, the blast result showed that *Hydra magnipapillata* and *Nematostella vectensis NF-κB* genes best matched with human *NFKB1* gene, but not *Rel* subfamily genes. Gene structure analysis showed that RHD domain and IPT domain of *Nematostella vectensis NF-κB* gene had same number of introns with mammal *NF-κB* subfamily, with 4 introns and 2 introns, respectively. Further analysis of intron phase showed that the intron phase was also similar with other *NF-κB* subfamily genes, with 0, 0, 2, 1 and 1, 0 in turn, respectively. However, these two genes had the highest identity and similarity to *Rel* subfamily genes (Table S4). Especially, the *Nematostella vectensis NF-κB* gene branch had undergone very strong positive selection (*ω* = 65.918) possibly due to the shift of gene structure and motifs (Fig S1). These structures and motifs change of *Hydra magnipapillata NF-κB* gene might be one of the reasons for these two genes belong to neither *Rel* subfamily nor *NF-κB* subfamily in the evolutionary history. We inferred that although the *Hydra magnipapillata* and *Nematostella vectensis NF-κB* genes best matched with *NF-κB* subfamily genes, it might be an ancestor gene of vertebrate *Rel* subfamily genes.

Interestingly, although components related to the insect and vertebrate TLR signaling pathways, including *TIR-1* (*SARM1*-like gene), *TRF-1* (*TRAF*-like gene), *PIK-1* (*Pelle* or *IRAK*-like gene), TOL-1 (*TLR*-like gene) and *IκB*-1 (*IκB*-like gene) [26], were identified in *Caenorhabditis elegans*, the TLR signaling pathway was regarded to be lost in *Caenorhabditis elegans* during the evolution history for no MyD88 scaffold protein and NF-κB-like transcription factor homologues in its genome. *Caenorhabditis elegans* might utilize other immune-related pathways and proteins to fight against pathogens, such as heat-shock transcription factor (HSF)-1 pathway [52], p38 MAP kinase Pathway [53], insulin signaling [54], [55], FSHR-1 [56], and so on.

Similar to other major pathways, the components and complexity of TLR signaling pathway increased from insect to mammal with the evolution of species and changed in the living environment. Firstly, the *TLR*, *IKK*, *IκB* and *NF-κB* genes underwent duplication events during their evolutionary history. This might have conferred more flexibility to the pathway as the various paralogs could allow fine-tuning of function in different organs [29]. Secondly, the adaptor molecular genes had been enlarged in the evolution history. Our analysis pointed out that the *MAL*-like gene and the *TRAM*-like gene were first detected in amphioxus; and the *TRIF* was first detected in vertebrate. The diversity of the toll-like receptors and the adaptor molecules might connect with recognition of pathogens. Thirdly, in addition to duplication event of *NF-κB* genes, the gene structure and conservation motif of *NF-κB* genes had shifted in the evolution history. For instances, *NF-*κ*B* genes in *Hydra magnipapillata* and *Nematostella vectensis* only contains RHD domain and IPT domain without any ANK repeats and Death domain and *Relish* gene in *Drosophila melanogaster* without coding Death domain has only two exons compared to other *NF-*κ*B* subfamily genes containing at least 18 exons (Figure 6).

In summary, we found that the selection constraint of TLR signaling pathway-related genes was negatively correlated with its position and all genes were highly conserved and underwent relatively strong purifying selection. The distribution of selective pressure along the pathway was driven by differential nonsynonymous substitution level. More importantly, we provided supporting evidences to show that the TLR signaling pathway might present in a common ancestor of sponges and eumetazoa, with gene duplication events, adaptor molecular enlarged, gene structure and conservation motif of *NF-κB* genes shifted in the their evolutionary history. In addition, *Hydra magnipapillata* and *Nematostella vectensis NF-κB* genes only contained RHD domain and IPT domain, but lost ANK repeats and Death domain; *Drosophila melanogaster Relish* gene lost Death domain and had only two exons, while other *NF-*κ*B* subfamily genes had at least 18 exons. Despite *NF-*κ*B* genes had strong selection pressure in the evolution, they had positive selection in some branches of the evolutionary tree. It might be connected with the genes duplication, genes structure shifted and domains lost. Therefore, the gene duplication might be connection with positive selection of specific branches. These results help us to understand the evolution history of TLR signal pathway and gene structure and conservation motif shifted of *NF-κB*. Our results are helpful for better understanding the selection constraint and evolutionary history of TLR signaling pathway.

## Supporting Information

Figure S1 Phylogenetic relationship of NF-κB family genes. All sequences were downloaded from NCBI, and aligned with MUSCLE. Trees were constructed as described in methods. The bootstrap consensus tree inferred from 1000 replicates. BI tree and ML tree have similar topology, with two clusters: *Rel* subfamily and *NF-κB* subfamily, respectively. The pentagram represents the branch underwent very strong positive selection in the evolutionary history.
(TIF)

Table S1 The distribution of TLR signaling pathway-related genes in different animals.
(DOC)

Table S2 Results of codon-based tests of selection.
(DOC)

Table S3 The gene ID. of NF-κB-mediated TLR signaling pathway in different organisms.
(DOC)

Table S4 The identity and similarity between any two NF-κB family genes in different organisms. The upper matrix is identity, and the lower matrix is similarity.
(DOC)
