# Supplementary material for: The Evolution and Origin of Animal Toll-Like Receptor Signaling Pathway Revealed by Network-Level Molecular Evolutionary Analyses
Source: PLoS One. 2012 Dec 7;7(12):e51657. doi: 10.1371/journal.pone.0051657 (PMC3517549; doi:10.1371/journal.pone.0051657)
Supplement: Table S1 — The distribution of TLR signaling pathway-related genes in different animals. (DOC) [file pone.0051657.s002.doc]

| genes | human | chimpanzee | Macaca | mouse | cow | chick | frog | zebrafish |
| --- | --- | --- | --- | --- | --- | --- | --- | --- |
| TLR1 |  |  |  |  |  |  |  |  |
| TLR2 |  |  |  |  |  |  |  |  |
| TLR3 |  |  |  |  |  |  |  |  |
| TLR4 |  |  |  |  |  |  | XP_002942581.1 |  |
| TLR5 |  |  |  |  |  |  |  |  |
| TLR6 |  |  |  |  |  | same to TLR1 | XP_002938703.1 | same to TLR1 |
| TLR7 |  |  |  |  |  |  |  |  |
| TLR8 |  |  |  |  |  | N |  |  |
| TLR9 |  |  |  |  |  | N | ENSXETP00000013094 |  |
| MyD88 | NP_001166038.1 |  |  |  |  |  |  |  |
| TIRAP |  |  |  |  |  |  |  |  |
| TRAM |  |  |  |  |  | N | N | N |
| TRIF |  |  |  |  |  |  | N |  |
| TOLLIP |  |  |  |  |  |  |  |  |
| IRAK1 |  |  |  |  |  | N |  |  |
| IRAK4 |  | XM_001166075.2 |  |  |  |  |  |  |
| TRAF6 |  |  |  |  |  |  |  |  |
| TRAF3 | ENSP00000332468 |  |  |  |  |  |  |  |
| TAB1 |  |  |  |  |  |  |  |  |
| TAB2 |  |  |  |  |  |  |  | wise2 |
| TAK1 |  |  |  |  |  |  |  |  |
| RIPK1 |  |  |  |  |  |  |  |  |
| IKKa |  |  |  |  |  |  |  |  |
| IKKb |  |  |  |  |  |  |  |  |
| IKKg |  | XP_003317847.1 |  |  |  | N |  |  |
| IKKe |  | XP_001146274.2 |  |  |  |  |  |  |
| TBK1 |  |  |  |  |  |  |  |  |
| MEK1 |  |  |  |  |  |  |  |  |
| MEK2 |  |  |  |  |  |  | N |  |
| MKK3 |  |  |  |  |  |  | N | N |
| MKK6 |  |  |  |  |  | N |  |  |
| MKK4 |  |  |  |  |  |  |  |  |
| MKK7 |  |  |  |  |  | N |  |  |
| IKBa |  |  |  |  |  |  |  |  |
| p105 |  |  |  |  |  |  |  | wise2 |
| p65 |  |  |  |  |  |  |  | NP_001001839.2 |
| MAPK1 |  |  |  |  |  |  |  |  |
| MAPK3 |  |  |  |  |  | N | N |  |
| MAPK11 |  | N |  |  |  |  |  |  |
| MAPK12 |  |  |  |  |  |  |  |  |
| MAPK13 |  |  |  |  |  |  |  |  |
| MAPK14 |  |  | wise2 |  |  |  |  |  |
| MAPK8 |  |  |  |  |  |  |  |  |
| MAPK9 | NP_620709.1 |  | EHH27119.1 |  |  |  | N |  |
| MAPK10 |  | XP_001155430.1 |  |  |  |  |  |  |
| IRF5 |  |  |  |  |  |  |  | ABY91289.1 |
| IRF7 |  |  |  |  |  |  |  |  |
| IRF3 |  |  |  |  |  | same to IRF7 |  |  |
| FOS |  |  |  |  |  |  |  |  |
| JUN |  |  |  |  |  |  |  |  |

Table S1. The distribution of TLR signaling pathway genes in different animals.

^#^*Note*:

N: the gene not present in detected animal.

wise2: the sequence was predicted with Wise2 program.

gene ID.: the sequence from NCBI or Ensmble.
