# Supplementary material for: The Evolution and Origin of Animal Toll-Like Receptor Signaling Pathway Revealed by Network-Level Molecular Evolutionary Analyses
Source: PLoS One. 2012 Dec 7;7(12):e51657. doi: 10.1371/journal.pone.0051657 (PMC3517549; doi:10.1371/journal.pone.0051657)
Supplement: Table S2 — Results of codon-based tests of selection. (DOC) [file pone.0051657.s003.doc]

|  | lnL (M1a) | lnL (M2a) | *2Δl* (M1a vs. M2a) | ln (M7) | ln (M8) | *2Δl* (M7 vs. M8) | M8a | *2Δl* (M8 vs. M8a) |
| --- | --- | --- | --- | --- | --- | --- | --- | --- |
| TLR1 | -12171.9 | -12171.9 | 0.0 | -12130.3 | -12123.5 | 13.6 ** | -12124.5 | 2.0 |
| TLR2 | -13419.2 | -13419.2 | 0.0 | -13355.8 | -13355.5 | 0.7 | -13355.8 | 0.7 |
| TLR3 | -14074.9 | -14074.9 | 0.0 | -13995.5 | -13993.5 | 4.0 | -13994.4 | 1.9 |
| TLR4 | -14894.0 | -14894.0 | 0.0 | -14854.5 | -14845.4 | 18.1 *** | -14847.7 | 4.6 * |
| TLR5 | -15193.7 | -15193.7 | 0.0 | -15125.9 | -15121.8 | 8.1 | -15123.3 | 3.0 |
| TLR6 | -8853.4 | -8853.4 | 0.0 | -8837.0 | -8836.8 | 0.5 | -8837.0 | 0.4 |
| TLR7 | -12873.0 | -12873.0 | 0.0 | -12839.3 | -12834.3 | 10.1 | -12836.3 | 3.9 * |
| TLR8 | -14359.9 | -14359.9 | 0.0 | -14339.1 | -14331.9 | 14.3 *** | -14332.4 | 1.0 |
| TLR9 | -15212.2 | -15212.2 | 0.0 | -15110.5 | -15102.4 | 16.3 *** | -15103.2 | 1.6 |
| MyD88 | -4109.0 | -4109.0 | 0.0 | -4085.1 | -4083.5 | 3.2 | -4083.5 | 0.0 |
| TIRAP | -3576.1 | -3576.1 | 0.0 | -3550.7 | -3547.5 | 6.5 * | -3548.7 | 2.5 |
| TRAM | -1799.2 | -1799.2 | 0.0 | -1798.6 | -1797.9 | 1.4 | -1798.6 | 1.4 |
| TRIF | -8922.1 | -8922.1 | 0.0 | -8879.2 | -8879.2 | 0.0 | -8879.2 | 0.1 |
| TOLLIP | -3510.8 | -3510.8 | 0.0 | -3485.5 | -3485.4 | 0.3 | -3485.4 | 0.0 |
| IRAK1 | -2310.8 | -2310.8 | 0.0 | -2313.0 | -2310.0 | 6.0 * | -2310.0 | 0.0 |
| IRAK4 | -4940.6 | -4940.6 | 0.0 | -4904.7 | -4903.3 | 2.8 | -4903.3 | 0.1 |
| TRAF6 | -7086.7 | -7086.7 | 0.0 | -7021.8 | -7019.9 | 3.8 | -7020.3 | 0.8 |
| TRAF3 | -6691.0 | -6691.0 | 0.0 | -6618.8 | -6618.8 | 0.0 | -6619.0 | 0.5 |
| TAB1 | -3505.9 | -3505.9 | 0.0 | -3483.8 | -3483.8 | 0.0 | -3483.8 | 0.1 |
| TAB2 | -5824.3 | -5824.3 | 0.0 | -5815.9 | -5814.7 | 2.3 | -5814.9 | 0.3 |
| TAK1 | -1637.4 | -1637.4 | 0.1 | -1631.0 | -1631.0 | 0.0 | -1631.0 | 0.0 |
| RIPK1 | -9810.6 | -9810.6 | 0.0 | -9747.3 | -9745.7 | 3.2 | -9746.7 | 1.9 |
| IKKa | -8560.9 | -8560.9 | 0.0 | -8514.0 | -8509.9 | 8.1 * | -8509.9 | 0.0 |
| IKKb | -6155.4 | -6155.4 | 0.0 | -6138.1 | -6136.6 | 3.0 | -6136.7 | 0.2 |
| IKKg | -4572.7 | -4572.7 | 0.0 | -4532.4 | -4532.3 | 0.2 | -4532.3 | 0.1 |
| IKKe | -4087.8 | -4087.8 | 0.0 | -4041.1 | -4038.1 | 6.1 * | -4040.1 | 4.0 * |
| TBK1 | -8907.1 | -8907.1 | 0.0 | -8843.1 | -8843.0 | 0.1 | -8843.3 | 0.5 |
| MEK1 | -4100.2 | -4100.2 | 0.0 | -4069.0 | -4069.0 | 0.0 | -4069.1 | 0.2 |
| MEK2 | -2849.7 | -2849.7 | 0.0 | -2838.9 | -2838.4 | 0.9 | -2838.4 | 0.1 |
| MKK3 | -1847.0 | -1847.0 | 0.0 | -1845.1 | -1845.1 | 0.0 | -1845.1 | 0.1 |
| MKK6 | -3082.8 | -3082.8 | 0.0 | -3070.4 | -3069.9 | 0.9 | -3070.0 | 0.1 |
| MKK4 | -3267.2 | -3266.8 | 0.6 | -3268.9 | -3260.6 | 16.8 *** | -3262.1 | 3.0 |
| MKK7 | -3601.4 | -3601.4 | 0.0 | -3574.4 | -3574.4 | 0.0 | -3574.3 | 0.2 |
| IkBa | -4256.9 | -4256.9 | 0.0 | -4221.5 | -4221.0 | 0.8 | -4221.2 | 0.2 |
| p105 | -8058.1 | -8058.1 | 0.0 | -8052.0 | -8043.5 | 17.0 *** | -8044.9 | 2.9 |
| p65 | -6254.8 | -6254.8 | 0.0 | -6197.9 | -6193.7 | 8.4 * | -6193.7 | 0.1 |
| MAPK1 | -3057.1 | -3057.8 | 1.3 | -3053.6 | -3053.6 | 0.0 | -3053.6 | 0.1 |
| MAPK3 | -2968.9 | -2968.9 | 0.0 | -2961.9 | -2960.1 | 3.7 | -2960.7 | 1.3 |
| MAPK11 | -4032.1 | -4032.1 | 0.0 | -3977.8 | -3977.8 | 0.0 | -3977.7 | 0.2 |
| MAPK12 | -4148.6 | -4148.6 | 0.0 | -4112.0 | -4112.0 | 0.0 | -4111.7 | 0.5 |
| MAPK13 | -4595.2 | -4595.2 | 0.0 | -4544.9 | -4544.9 | 0.0 | -4544.9 | 0.1 |
| MAPK14 | -3709.4 | -3709.4 | 0.0 | -3679.1 | -3679.1 | 0.0 | -3679.4 | 0.5 |
| MAPK8 | -3834.9 | -3833.8 | 2.2 | -3835.0 | -3823.5 | 23.0 *** | -3826.2 | 5.4 * |
| MAPK9 | -2505.8 | -2505.8 | 0.0 | -2492.4 | -2492.4 | 0.0 | -2492.4 | 0.0 |
| MAPK10 | -3495.6 | -3495.6 | 0.0 | -3495.5 | -3492.0 | 7.1 | -3491.9 | 0.1 |
| IRF5 | -4303.6 | -4303.6 | 0.0 | -4285.3 | -4283.3 | 4.0 | -4283.4 | 0.2 |
| IRF7 | -6978.6 | -6978.6 | 0.0 | -6936.7 | -6927.7 | 18.1 *** | -6929.0 | 2.6 |
| IRF3 | -4221.6 | -4221.6 | 0.0 | -4203.3 | -4202.7 | 1.1 | -4203.2 | 1.0 |
| FOS | -4387.6 | -4387.6 | 0.0 | -4336.8 | -4336.8 | 0.0 | -4336.7 | 0.2 |
| JUN | -2904.5 | -2904.5 | 0.0 | -2866.4 | -2866.4 | 0.0 | -2866.1 | 0.6 |

Table S2. Results of codon-based tests of selection.

Note: The codon-based test of selection was performed by codeml software in PAML 4.4 package. *, *P* < 0.05, **, *P* < 0.01, ***, *P* < 0.001.
