# Supplementary material for: The Evolution and Origin of Animal Toll-Like Receptor Signaling Pathway Revealed by Network-Level Molecular Evolutionary Analyses
Source: PLoS One. 2012 Dec 7;7(12):e51657. doi: 10.1371/journal.pone.0051657 (PMC3517549; doi:10.1371/journal.pone.0051657)
Supplement: Table S3 — The gene ID. of NF-κB-mediated TLR signaling pathway in different organisms. (DOC) [file pone.0051657.s004.doc]

Table S3. The gene ID. of NF-қB-mediated TLR signaling pathway in different organisms.

| Species | genes | mRNA → protein |
| --- | --- | --- |
| Homo sapiens | TLR1 | NM_003263.3 → NP_003254.2 |
| Homo sapiens | TLR2 | NM_003264.3 → NP_003255.2 |
| Homo sapiens | TLR3 | NM_003265.2 → NP_003256.1 |
| Homo sapiens | TLR4 | NM_003266.3 → NP_003257.1 |
| Homo sapiens | TLR5 | NM_003268.5 → NP_003259.2 |
| Homo sapiens | TLR6 | NM_006068.4 → NP_006059.2 |
| Homo sapiens | TLR7 | NM_016562.3 → NP_057646.1 |
| Homo sapiens | TLR8 | NM_138636.4 → NP_619542.1 |
| Homo sapiens | TLR9 | NM_017442.3 → NP_059138.1 |
| Homo sapiens | TLR10 | NM_001017388.2 → NP_001017388.1 |
| Homo sapiens | MyD88 | NM_001172567.1 → NP_001166038.1 |
| Homo sapiens | SARM1 | NM_015077.2 → NP_055892.2 |
| Homo sapiens | TRAM(TICAM2) | NM_021649.6 → NP_067681.1 |
| Homo sapiens | TRIF(TICAM1) | NM_182919.3 → NP_891549.1 |
| Homo sapiens | TIRAP(MAL) | NM_148910.2 → NP_683708.1 |
| Homo sapiens | IRAK1 | NM_001569.3 → NP_001560.2 |
| Homo sapiens | IRAK4 | NM_001114182.2 → NP_001107654.1 |
| Homo sapiens | TRAF6 | NM_004620.3 → NP_004611.1 |
| Homo sapiens | TAB1 | NM_006116.2 → NP_006107.1 |
| Homo sapiens | TAB2 | NM_015093.4 → NP_055908.1 |
| Homo sapiens | TAK1(MAP3K7) | NM_145331.1 → NP_663304.1 |
| Homo sapiens | IKK | NM_001278.3 → NP_001269.3 (IKKa) |
| Homo sapiens |  | NM_001556.2 → NP_001547.1 (IKKb) |
| Homo sapiens |  | NM_014002.3 → NP_054721.1 (IKKe) |
| Homo sapiens |  | NM_001099856.2 → NP_001093326.2 (IKKg) |
| Homo sapiens | IқB | NM_020529.2 → NP_065390.1 (IқBa) |
| Homo sapiens |  | NM_001243116.1 → NP_001230045.1 (IkBb) |
| Homo sapiens |  | NM_004556.2 → NP_004547.2 (IkBe) |
| Homo sapiens |  | NM_031419.3 → NP_113607.1 (NFKBIZ) |
| Homo sapiens |  | NM_139239.1 → NP_640332.1 (NFKBID) |
| Homo sapiens | NF-қB | NM_021975.3 → NP_068810.3 (RELA) |
| Homo sapiens |  | NM_006509.3 → NP_006500.2 (RELB) |
| Homo sapiens |  | NM_002908.2 → NP_002899.1 (REL) |
| Homo sapiens |  | NM_003998.3 → NP_003989.2 (NF-қB1) |
| Homo sapiens |  | NM_001077494.1 → NP_001070962.1 (NF-қB2) |
| Xenopus tropicalis | TLR1 | XM_002938649.1 → XP_002938695.1 |
| Xenopus tropicalis | TLR2 | XM_002933491.1 → XP_002933537.1 |
| Xenopus tropicalis | TLR3 | XM_002934402.1 → XP_002934448.1 |
| Xenopus tropicalis | TLR4 | XM_002942535.1 → XP_002942581.1 |
| Xenopus tropicalis | TLR5 | XM_002940696.1 → XP_002940742.1 |
| Xenopus tropicalis | TLR6 | XM_002938657.1 → XP_002938703.1 |
| Xenopus tropicalis | TLR7 | XM_002936487.1 → XP_002936533.1 |
| Xenopus tropicalis | TLR8 | XM_002933813.1 → XP_002933859.1 |
| Xenopus tropicalis | TLR10 | XM_002943049.1 → XP_002943095.1 |
| Xenopus tropicalis | TLR12 | XM_002941824.1 → XP_002941870.1 |
| Xenopus tropicalis | TLR13 | XM_002935001.1 → XP_002935047.1 |
| Xenopus tropicalis | MyD88 | NM_001016837.2 → NP_001016837.1 |
| Xenopus tropicalis | SARM1 | XM_002937143.1 → XP_002937189.1 |
| Xenopus tropicalis | TRAM(TICAM2) | None |
| Xenopus tropicalis | TRIF(TICAM1) | None |
| Xenopus tropicalis | TIRAP(MAL) | NM_001044460.1 → NP_001037925.1 |
| Xenopus tropicalis | IRAK1 | NM_001006712.1 → NP_001006713.1 |
| Xenopus tropicalis | IRAK4 | NM_001123405.1 → NP_001116877.1 |
| Xenopus tropicalis | TRAF6 | NM_001008161.2 → NP_001008162.2 |
| Xenopus tropicalis | TAB1 | NM_001016463.2 → NP_001016463.1 |
| Xenopus tropicalis | TAB2 | NM_001097294.1 → NP_001090763.1 |
| Xenopus tropicalis | TAK1(MAP3K7) | NM_001100261.1 → NP_001093731.1 |
| Xenopus tropicalis | IKK | NM_001016900.2 → NP_001016900.1 |
| Xenopus tropicalis |  | NM_001005651.1 → NP_001005651.1 |
| Xenopus tropicalis |  | XM_002940511.1 → XP_002940557.1 |
| Xenopus tropicalis |  | NM_001126749.1 → NP_001120221.1 |
| Xenopus tropicalis | IқB | NM_001004851.1 → NP_001004851.1 |
| Xenopus tropicalis |  | NM_001126918.1 → NP_001120390.1 |
| Xenopus tropicalis |  | NM_001130266.1 → NP_001123738.1 |
| Xenopus tropicalis | NF-қB | NM_001001211.1 → NP_001001211.1 |
| Xenopus tropicalis |  | XM_002932608.1 → XP_002932654.1 |
| Xenopus tropicalis |  | NM_001102707.1 → NP_001096177.1 |
| Xenopus tropicalis |  | XM_002934759.1 → XP_002934805.1 |
| Xenopus tropicalis |  | NM_001005032.1 → NP_001005032.1 |
| Danio rerio | TLR1 | NM_001130593.1 → NP_001124065.1 |
| Danio rerio | TLR2 | NM_212812.1 → NP_997977.1 |
| Danio rerio | TLR3 | NM_001013269.2 → NP_001013287.2 |
| Danio rerio | TLR4 | XM_001919664.3 → XP_001919699.3 |
| Danio rerio | TLR5 | NM_001130595.1 → NP_001124067.1 |
| Danio rerio | TLR7 | XM_003199261.1 → XP_003199309.1 |
| Danio rerio | TLR8 | XM_002665908.2 → XP_002665954.2 |
| Danio rerio | TLR9 | NM_001130594.1 → NP_001124066.1 |
| Danio rerio | TLR13 | XM_001344652.4 → XP_001344688.4 |
| Danio rerio | TLR18 | NM_001089350.1 → NP_001082819.1 |
| Danio rerio | TLR19 | XM_002664846.2 → XP_002664892.2 |
| Danio rerio | TLR20 | XM_003199232.1 → XP_003199280.1 |
| Danio rerio | TLR21 | NM_001199335.1 → NP_001186264.1 |
| Danio rerio | TLR22 | NM_001128675.1 → NP_001122147.1 |
| Danio rerio | MyD88 | NM_212814.2 → NP_997979.2 |
| Danio rerio | SARM1 | NM_001130596.1 → NP_001124068.1 |
| Danio rerio | TRAM(TICAM2) | None |
| Danio rerio | TRIF(TICAM1) | NM_001044759.1 → NP_001038224.1 |
| Danio rerio | TIRAP(MAL) | XM_002667112.2 → XP_002667158.2 |
| Danio rerio | IRAK1 | CAP19555.1 (Genebank protein ID.) |
| Danio rerio | IRAK4 | AY616584.1 → AAT37635.1 |
| Danio rerio | TRAF6 | NM_001044752.1 → NP_001038217.1 |
| Danio rerio | TAB1 | BC058295.1 → AAH58295.1 |
| Danio rerio | TAB2 | Q5RFW2.1 (UniProtKB/Swiss-Prot protein ID.) |
| Danio rerio | TAK1(MAP3K7) | NM_001020750.1 → NP_001018586.1 |
| Danio rerio | IKK | NM_200317.1 → NP_956611.1 |
| Danio rerio |  | NM_001123265.1 → NP_001116737.1 |
| Danio rerio |  | NM_001002751.1 → NP_001002751.1 |
| Danio rerio |  | NM_001014344.1 → NP_001014366.1 |
| Danio rerio | IқB | NM_213184.1 → NP_998349.1 |
| Danio rerio |  | NM_001128795.1 → NP_001122267.1 |
| Danio rerio |  | NM_001080089.1 → NP_001073558.1 |
| Danio rerio | NF-қB | NM_001001839.2 → NP_001001839.2 |
| Danio rerio |  | XM_001335557.1 → XP_001335593.2 |
| Danio rerio |  | NM_001001841.2 → NP_001001841.2 |
| Danio rerio |  | XM_003199975.1 → XP_003200023.1 |
| Danio rerio |  | NM_001001840.2 → NP_001001840.2 |
| Branchiostoma floridae | TLR6-like | XM_002601335.1 → XP_002601381.1; best match human TLR6 |
| Branchiostoma floridae | TLR4a-like | XM_002594025.1 → XP_002594071.1; best match human TLR4 |
| Branchiostoma floridae | TLR3a-like | XM_002591438.1 → XP_002591484.1; best match human TLR3 |
| Branchiostoma floridae | TLR5a-like | XM_002607903.1 → XP_002607949.1; best match human TLR5 |
| Branchiostoma floridae | TLR7-like | XM_002611143.1 → XP_002611189.1; best match human TLR7 |
| Branchiostoma floridae | TLR4b-like | XM_002611737.1 → XP_002611783.1; best match human TLR4 |
| Branchiostoma floridae | TLR9-like | XM_002601649.1 → XP_002601695.1; best match human TLR9 |
| Branchiostoma floridae | TLR1-like | XM_002611892.1 → XP_002611938.1; best match human TLR1 |
| Branchiostoma floridae | TLR5b-like | XM_002590661.1 → XP_002590707.1; best match human TLR5 |
| Branchiostoma floridae | TLR4c-like | XM_002590620.1 → XP_002590666.1; best match human TLR4 |
| Branchiostoma floridae | TLR3b-like | XM_002590143.1 → XP_002590189.1; best match human TLR3 |
| Branchiostoma floridae | TLR5c-like | XM_002590621.1 → XP_002590667.1; best match human TLR5 |
| Branchiostoma floridae | TLR5d-like | XM_002589802.1 → XP_002589848.1; best match human TLR5 |
| Branchiostoma floridae | TLR1b-like | XM_002590659.1 → XP_002590705.1; best match human TLR1 |
| Branchiostoma floridae | TLR2-like | XM_002590662.1 → XP_002590708.1; best match human TLR2 |
| Branchiostoma floridae | MyD88 | XM_002592077.1 → XP_002592123.1 |
| Branchiostoma floridae | SARM1 | XM_002587493.1 → XP_002587539.1 |
| Branchiostoma floridae | TRAM(TICAM2) | XM_002601530.1 → XP_002601576.1 |
| Branchiostoma floridae | TRIF(TICAM1) | None |
| Branchiostoma floridae | TIRAP(MAL) | BW729041.1 (EST) |
| Branchiostoma floridae | IRAK1 | XM_002606536.1 → XP_002606582.1; best match human IRAK1 |
| Branchiostoma floridae | IRAK4 | XM_002601673.1 → XP_002601719.1; best match human IRAK4 |
| Branchiostoma floridae | TRAF6 | XM_002592106.1 → XP_002592152.1; best match human TRAF2 |
| Branchiostoma floridae | TAB1 | XM_002595563.1 → XP_002595609.1 |
| Branchiostoma floridae | TAB2 | XM_002604238.1 → XP_002604284.1 |
| Branchiostoma floridae | TAK1(MAP3K7) | XM_002589723.1 → XP_002589769.1 |
| Branchiostoma floridae | IKK | XM_002604684.1 → XP_002604730.1; best match human IKKa |
| Branchiostoma floridae |  | XM_002601820.1 → XP_002601866.1; best match human IKKg |
| Branchiostoma floridae | IқB | XM_002591941.1 → XP_002591987.1 |
| Branchiostoma floridae | NF-қB | XM_002613102.1 → XP_002613148.1 |
| Branchiostoma floridae |  | p105 (UCSC, wise2) |
| Drosophila melanogaster | Toll-9 | NM_140957.3 → NP_649214.1; best match human TLR1 |
| Drosophila melanogaster | Tollo | NM_080018.1 → NP_524757.1; best match human TLR7 |
| Drosophila melanogaster | Toll | NM_170287.1 → NP_733166.1; best match human TLR8 |
| Drosophila melanogaster | Toll-6 | NM_079357.2 → NP_524081.1; best match human TLR4 |
| Drosophila melanogaster | Toll-7 | NM_079073.2 → NP_523797.1; best match human TLR7 |
| Drosophila melanogaster | Tehao | NM_058090.2 → NP_477438.1; best match human TLR8 |
| Drosophila melanogaster | 18w | NM_057466.2 → NP_476814.1; best match human TLR8 |
| Drosophila melanogaster | Msprox | NM_141462.2 → NP_649719.2; best match human TLR9 |
| Drosophila melanogaster | Toll-4 | NM_078795.2 → NP_523519.2; best match human TLR5 |
| Drosophila melanogaster | MyD88 | NM_136635.2 → NP_610479.1 |
| Drosophila melanogaster | Ect4 | NM_001104071.1 → NP_001097541.1; best match human SARM1 |
| Drosophila melanogaster | TRAM(TICAM2) | None |
| Drosophila melanogaster | TRIF(TICAM1) | None |
| Drosophila melanogaster | TIRAP(MAL) | None |
| Drosophila melanogaster | PLL | NM_057623.3 → NP_476971.1; best match human IRAK1 |
| Drosophila melanogaster | IRAK4 | None |
| Drosophila melanogaster | TRAF6 | NM_078525.3 → NP_511080.2 |
| Drosophila melanogaster | TAB1 | None |
| Drosophila melanogaster | TAB2 | NM_137564.2 → NP_611408.2 |
| Drosophila melanogaster | TAK1(MAP3K7) | NM_079356.2 → NP_524080.1 |
| Drosophila melanogaster | IK2 | NM_165337.1 → NP_724278.1; best match human IKKe |
| Drosophila melanogaster | ird5 | NM_080012.3 → NP_524751.2; best match human IKKb |
| Drosophila melanogaster | key | NM_079132.2 → NP_523856.2; |
| Drosophila melanogaster | cactus | NM_165152.1 → NP_723960.1; best match human IkBa |
| Drosophila melanogaster | dl | NM_165219.1 → NP_724054.1; best match human REL |
| Drosophila melanogaster | dif | NM_001169527.1 → NP_001162998.1; best match human REL |
| Drosophila melanogaster | relish | NM_057746.3 → NP_477094.1; best match human NF-kB1 |
| Caenorhabditis elegans | TOL-1 | NM_001025812.2 → NP_001020983.1 |
| Caenorhabditis elegans | MyD88 | None |
| Caenorhabditis elegans | TIR-1 | NM_065384.3 → NP_497785.1;best match human SARM1 |
| Caenorhabditis elegans | TRAM(TICAM2) | None |
| Caenorhabditis elegans | TRIF(TICAM1) | None |
| Caenorhabditis elegans | TIRAP(MAL) | None |
| Caenorhabditis elegans | PIK-1 | NM_070186.3 → NP_502587.2; best match human IRAK1 |
| Caenorhabditis elegans | IRAK4 | None |
| Caenorhabditis elegans | TRF-1 | NM_067372.3 → NP_499773.2; best match human TRAF6 |
| Caenorhabditis elegans | TAP-1 | NM_078027.3 → NP_510428.1; best match human TAB1 |
| Caenorhabditis elegans | TAB2 | None |
| Caenorhabditis elegans | TAK1(MAP3K7) | None |
| Caenorhabditis elegans | IқB-1 | NM_060174.4 → NP_492575.1; best match human IkBe |
| Hydra magnipapillata | TRR-1 | XM_002169069.1 → XP_002169105.1; best match human TLR4 |
| Hydra magnipapillata | TRR-2 | XM_002161544.1 → XP_002161580.1; best match human TLR2 |
| Hydra magnipapillata | MyD88 | None |
| Hydra magnipapillata | SARM1 | None |
| Hydra magnipapillata | TRAM(TICAM2) | None |
| Hydra magnipapillata | TRIF(TICAM1) | None |
| Hydra magnipapillata | TIRAP(MAL) | None |
| Hydra magnipapillata | IRAK1 | None |
| Hydra magnipapillata | IRAK4 | None |
| Hydra magnipapillata | TRAF6 | XM_002167513.1 → XP_002167549.1 (TRAF6, patial) |
| Hydra magnipapillata | TAB1 | XM_002166531.1 → XP_002166567.1 |
| Hydra magnipapillata | TAK1(MAP3K7) | XM_002162102.1 → XP_002162138.1 |
| Hydra magnipapillata | IKK | XM_002168469.1 → XP_002168505.1; best match human IKKb |
| Hydra magnipapillata |  | XM_002162703.1 → XP_002162739.1; best match human IKKe |
| Hydra magnipapillata |  | XM_002168380.1 → XP_002168416.1; best match human IKKg |
| Hydra magnipapillata | NF-қB | HQ660069.1 → ADU79237.1; best match human NF-kB1 |
| Nematostella vectensis | TLR1-like | XM_001629440.1 → XP_001629490.1; best match human TLR1 |
| Nematostella vectensis | TLR4-like | XM_001637924.1 → XP_001637974.1; best match human TLR4 |
| Nematostella vectensis | MyD88 | XM_001640955.1 → XP_001641005.1 |
| Nematostella vectensis | SARM1 | XM_001626973.1 → XP_001627023.1 |
| Nematostella vectensis | TRAM(TICAM2) | None |
| Nematostella vectensis | TRIF(TICAM1) | None |
| Nematostella vectensis | TIRAP(MAL) | None |
| Nematostella vectensis | IRAK1 | None |
| Nematostella vectensis | IRAK4 | None |
| Nematostella vectensis | TRAF6 | XM_001640786.1 → XP_001640836.1 |
| Nematostella vectensis | TAB1 | XM_001640311.1 → XP_001640361.1 |
| Nematostella vectensis | TAB2 | XM_001630587.1 → XP_001630637.1 |
| Nematostella vectensis | TAK1(MAP3K7) | XM_001639157.1 → XP_001639207.1 |
| Nematostella vectensis | IKK | HM754644.1 → ADQ57374.1 |
| Nematostella vectensis | IқB | EU092641.1 → ABU48531.1 |
| Nematostella vectensis | NF-қB | XM_001624973.1 → XP_001625023.1; best match human NF-kB1 |
| Amphimedon queenslandica | TLR6-like | XM_003386499.1 → XP_003386547.1; best match human TLR6 |
| Amphimedon queenslandica | TLR1-like | XM_003383366.1 → XP_003383414.1; best match human TLR1 |
| Amphimedon queenslandica | MyD88 | XM_003385562.1 → XP_003385610.1 |
| Amphimedon queenslandica | SARM1 | XM_003387071.1 → XP_003387119.1 |
| Amphimedon queenslandica | TRAM(TICAM2) | None |
| Amphimedon queenslandica | TRIF(TICAM1) | None |
| Amphimedon queenslandica | TIRAP(MAL) | None |
| Amphimedon queenslandica | IRAK1 | XM_003382696.1 → XP_003382744.1 |
| Amphimedon queenslandica | IRAK4 | XM_003388709.1 → XP_003388757.1 |
| Amphimedon queenslandica | TRAF6 | XM_003383497.1 → XP_003383545.1 |
| Amphimedon queenslandica | TAB1 | XM_003386838.1 → XP_003386886.1 |
| Amphimedon queenslandica | TAB2 | None |
| Amphimedon queenslandica | TAK1(MAP3K7) | XM_003390199.1 → XP_003390247.1 |
| Amphimedon queenslandica | IKK | XM_003382976.1 → XP_003383024.1 |
| Amphimedon queenslandica | IқB | XM_003387518.1 → XP_003387566.1 |
| Amphimedon queenslandica | NF-қB | XM_003382593.1 → XP_003382641.1 |
