# Supplementary material for: The Evolution and Origin of Animal Toll-Like Receptor Signaling Pathway Revealed by Network-Level Molecular Evolutionary Analyses
Source: PLoS One. 2012 Dec 7;7(12):e51657. doi: 10.1371/journal.pone.0051657 (PMC3517549; doi:10.1371/journal.pone.0051657)
Supplement: Table S4 — The identity and similarity between any two NF-κB family genes in different organisms. The upper matrix is identity, and the lower matrix is similarity. (DOC) [file pone.0051657.s005.doc]

Table S4. The identity and similarity between any two NF-қB family gene in different organisms. The upper matrix is identity, and the lower matrix is similarity.

|  | 1 | 2 | 3 | 4 | 5 | 6 | 7 | 8 | 9 | 10 | 11 | 12 | 13 |
| --- | --- | --- | --- | --- | --- | --- | --- | --- | --- | --- | --- | --- | --- |
| 1. *H. sapiens* RELA |  | 32.2 | 38.2 | 22.3 | 25.6 | 25.9 | 25.0 | 23.2 | 20.9 | 19.7 | 24.1 | 25.7 | 20.7 |
| 2. *H. sapiens* RELB | 49.6 |  | 27.8 | 20.4 | 21.7 | 25.6 | 19.8 | 20.5 | 20.2 | 20.3 | 20.2 | 26.3 | 17.9 |
| 3. *H. sapiens* REL | 55.4 | 46.7 |  | 23.3 | 25.1 | 24.3 | 27.4 | 23.4 | 21.6 | 20.9 | 22.7 | 23.2 | 23.7 |
| 4. *H. sapiens* NFKB1 | 31.7 | 30 | 35.0 |  | 42.2 | 14.2 | 41.4 | 25.1 | 22.4 | 23.7 | 16.6 | 21.5 | 33.6 |
| 5. *H. sapiens* NFKB2 | 35.4 | 33.2 | 38.0 | 57.4 |  | 15.0 | 37.9 | 24.3 | 24.2 | 24.0 | 17.8 | 21.2 | 33.8 |
| 6. *B. floridae* REL | 35.4 | 33.3 | 31.5 | 18.7 | 20.6 |  | 15.2 | 15.1 | 12.2 | 10.3 | 19.2 | 31.5 | 12.5 |
| 7. *B. floridae* NFKB1 | 37.4 | 32.2 | 40.1 | 54.7 | 55 | 21.6 |  | 23.6 | 20.7 | 22.8 | 17.2 | 24.3 | 33.4 |
| 8. *D. melanogaster* DL | 32.4 | 30.7 | 34.4 | 42.7 | 41.5 | 19.5 | 37.9 |  | 38.5 | 22.4 | 15.3 | 15.7 | 24.0 |
| 9. *D. melanogaster* Dif | 30.4 | 30.4 | 34.5 | 41.6 | 39.0 | 17.7 | 34.0 | 56.4 |  | 23.0 | 14.6 | 15.1 | 21.0 |
| 10. *D. melanogaster* Relish | 30.8 | 30.0 | 32.9 | 44.8 | 42.5 | 16.1 | 40.2 | 41.4 | 41.7 |  | 15.6 | 14.8 | 22.0 |
| 11. *H. magnipapillata* NFKB | 42.3 | 36.8 | 39.1 | 26.7 | 29.3 | 32.3 | 28.5 | 24.6 | 25.0 | 25.2 |  | 23.3 | 14.7 |
| 12. *N. vectensis* NFKB | 42.5 | 41.8 | 37.0 | 28.7 | 29.1 | 41.4 | 32.7 | 23.9 | 23.6 | 23.7 | 41.7 |  | 18.3 |
| 13. *A. queenslandica* NFKB | 30.2 | 26.3 | 33.7 | 51.3 | 48.1 | 17.0 | 47.1 | 39.2 | 39.0 | 41.6 | 24.6 | 25.4 |  |
